# Efficacy and Safety of Direct Oral Anticoagulants in Patients With Antiphospholipid Syndrome: A Systematic Review and Meta-Analysis

**DOI:** 10.7759/cureus.29449

**Published:** 2022-09-22

**Authors:** Keerthi Gullapalli, Rohan M Prasad, Abdullah Al-abcha, Zahin Hussain, Aseel Alsouqi, Osama Mosalem, Borys Hrinczenko

**Affiliations:** 1 Internal Medicine, Michigan State University-Sparrow Hospital, Lansing, USA; 2 Internal Medicine, Michigan State University College of Human Medicine, Lansing, USA; 3 Hematology and Oncology, University of Pittsburgh Medical Center, Pittsburgh, USA; 4 Hematology and Oncology, Michigan State University, Lansing, USA

**Keywords:** direct oral anticoagulants, vitamin k antagonists, antiphospholipid syndrome, thromboembolism, warfarin

## Abstract

Due to a high risk of recurrent thromboembolism in patients with antiphospholipid syndrome (APS), long-term anticoagulation is recommended. For decades, vitamin K antagonists (VKAs) have been the gold standard for thromboprophylaxis in these patients. Due to the widespread use of direct oral anticoagulants (DOACs) in various thromboembolic conditions and their potential advantages compared to VKAs, several studies have been conducted to evaluate their safety and efficacy in APS.

We performed a literature search using PubMed, Embase, and Cochrane databases for studies comparing DOACs to VKAs in patients with APS. Relative risk (RR) and the corresponding 95% confidence intervals (95% CI) were estimated for recurrent thromboembolic events, bleeding, and mortality.

A total of 1437 patients pooled from 12 studies were analyzed. The risk of recurrent thrombosis, especially arterial thrombosis, doubled with DOACs compared to VKAs (RR 2.61, 95% CI 1.44-4.71; p=0.001). The risk further increased in patients with a triple-positive antiphospholipid antibody profile (RR 4.50, 95% CI 1.91-10.63; p=0.0006) and with the use of rivaroxaban (RR 1.95, 95% CI 1.10-3.45; p=0.02). The risk of major bleeding and mortality were not significantly different between the two arms. A trend favoring DOACs compared to VKAs was observed for all bleeding events.

This meta-analysis comes in agreement with previous studies and supports the use of VKAs in APS. Our study revealed that VKAs remain the gold standard for the management of APS, especially triple-positive APS. DOACs, particularly rivaroxaban, are not as effective in preventing recurrent thromboembolism in high-risk APS patients. Further studies are needed to evaluate the role of DOACs apart from rivaroxaban with a focus on their efficacy in the management of isolated or double-positive APS.

## Introduction and background

Antiphospholipid syndrome (APS) is an autoimmune disorder characterized by at least one thromboembolic (TE) event (venous, arterial, or small vessel) and/or pregnancy morbidity (one or more unexplained fetal deaths, one or more premature births, and three or more unexplained consecutive spontaneous abortions) in the presence of at least one persistent (12 weeks) antiphospholipid antibody (aPL): lupus anticoagulant (LA), IgG or IgM anticardiolipin antibodies (aCL), IgG or IgM anti-β2-glycoprotein antibodies (anti- β2GPI) (Sapporo criteria) [1]. Patients who exhibit positive testing for all three antibodies (triple-positivity) appear to have a worse prognosis due to their high risk for recurrent thrombosis and pregnancy complications [1,2]. The estimated incidence of APS is approximately five per 100,000 persons per year, with a prevalence of around 40-50 cases per 100,000 persons, and is seen more commonly in women (1:3.5, male-female ratio) between 15-50 years of age [3]. Catastrophic APS, the most severe form of APS, which accounts for approximately 1% of all APS cases, is associated with an overall mortality rate of 37% [4].

After a first thrombotic event, the risk of recurrent thromboembolism increases by 10-67% in APS, and long-term anticoagulation is indicated [5,6]. For decades, vitamin K antagonists (VKAs) have been recommended as the gold standard agents for the treatment and prevention of recurrent TE events in APS. However, long-term treatment with VKAs is a great clinical challenge due to the need for close international normalized ratio (INR) monitoring, inconsistent quality of anticoagulation, lack of adherence, and the risk of major bleeding [5,6]. Direct oral anticoagulants (DOACs) emerged over the last decade as a practicable alternative to VKAs and have been widely used to treat and prevent several TE conditions; thanks to their capability to evade most obstacles that are seen with the use of VKAs as mentioned above [7].

Several clinical studies have previously evaluated the use of DOACs, predominantly rivaroxaban, in patients with APS, but the data on their safety and efficacy are conflicting [8-14]. A meta-analysis of these studies by Koval et al. [15] revealed an increased TE risk with DOACs compared with VKAs. More recently, multiple randomized and non-randomized studies have been conducted. Apixaban for secondary prevention of thromboembolism among patients with antiphospholipid syndrome (ASTRO-APS) is a randomized clinical trial (RCT) by Woller et al. that compared apixaban with warfarin in the treatment of APS [16]. The results revealed an increased number of recurrent thrombotic events in the DOACs arm (6 of 23) compared to warfarin (0 of 25). Pengo et al. [9] conducted the trial of rivaroxaban in antiphospholipid syndrome (TRAPS), an RCT that terminated prematurely on January 28, 2018, after finding a high incidence of arterial thrombosis in the rivaroxaban group. Following termination of the trial, most patients (n=109) were switched to warfarin, whereas six patients remained on DOACs. A two-year follow-up study describing the events between January 28, 2018, and January 28, 2020, was recently published, the results of which further support the use of warfarin in high-risk patients with APS [17]. Three retrospective studies further compared DOACs with warfarin in non-triple-positive APS patients [18-20]. Observations from Williams et al. [18] suggest that rivaroxaban might pose an increased risk for recurrent thromboembolism in these patients. In contrast, contrary data from Franke et al. [19] and Liu et al. [20] indicate that DOACs may be safe in this group. Due to contrasting results from different studies and the availability of several more recent RCTs and observational studies, we performed a systematic review and meta-analysis to compare the safety and efficacy of DOACs to VKAs in APS.

## Review

Materials and methods

Information Sources, Search Strategies, and Data Extraction

We performed a comprehensive literature search using multiple electronic databases: PubMed, Embase, and Cochrane, from inception to June 14, 2022. The search included the following keywords: “direct oral anticoagulants,” “apixaban,” “rivaroxaban," “dabigatran," “edoxaban,” and “vitamin K antagonists,” “warfarin,” and “antiphospholipid syndrome.” After removing duplicates, two reviewers (KG and RP) independently reviewed the search results and screened the articles against the inclusion and exclusion criteria to assess their eligibility.

Inclusion/Exclusion Criteria

The inclusion criteria consisted of (1) double-arm longitudinal studies; RCTs, and observational studies (prospective or retrospective), (2) comparison of DOACs versus VKAs or warfarin, (3) DOACs were either rivaroxaban, apixaban, dabigatran, or edoxaban, (4) reported either efficacy or safety outcomes; recurrent thromboembolic events, major bleeding, any bleeding, and mortality, (5) human subjects, and (6) adults diagnosed with APS. Exclusion criteria consisted of (1) ongoing or irretrievable data, (2) single-arm studies, (3) animal studies, (4) case reports, case series, reviews, abstracts, protocols, letters to the editor, comments, or summaries for patients, (5) studies with an unclear outcome or conclusion, and (6) articles published in a language other than English.

Outcome

The primary outcome of this meta-analysis was recurrent TE events, which may include a composite of arterial and venous events. Arterial events include stroke, transient ischemic attack (TIA), myocardial infarction (MI), or peripheral artery disease (PAD). Venous events include deep vein thrombosis (DVT), pulmonary embolism (PE), cerebral venous thrombosis (CVT), or recurrent thrombosis in an inferior vena cava (IVC) filter. Secondary outcomes included major bleeding (as defined by the International Society on Thrombosis and Hemostasis criteria), all bleeding events (including major bleeding, clinically relevant non-major bleeding, and minor bleeding), and mortality.

Data Analysis

We scrutinized results from each study using the intention-to-treat (ITT) method when available. Data were pooled using Review Manager (RevMan) Version 5.4.1 (The Cochrane Collaboration, Denmark, 2014). The outcomes were treated as dichotomous variables. The Mantel-Haenszel random-effects models were used to estimate the risk ratios (RR) and the corresponding 95% confidence intervals (95% CI). Two-sided p values <0.05 were considered statistically significant. Heterogeneity was assessed using I^2^. Statistical heterogeneity was considered substantial if I^2^ >50%. The meta-analysis was performed in accordance with the Preferred Reporting Items for Systematic Reviews and Meta-Analysis (PRISMA) guidelines [21]. Each study was evaluated independently by KG and RP to evaluate the risk of bias. 

Results

Included Studies

**Figure 1:**
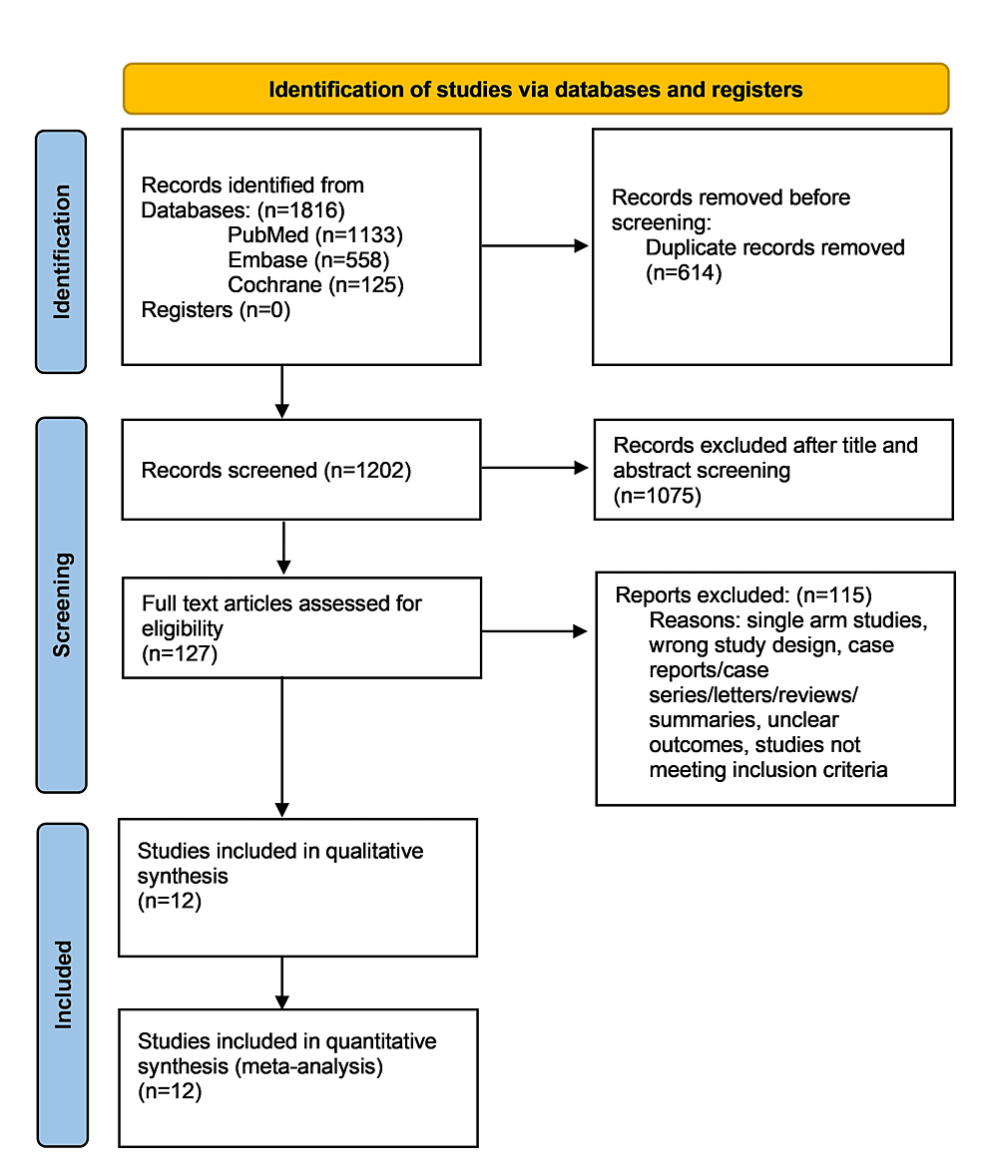
PRISMA flow chart. PRISMA: preferred reporting items for systematic reviews and meta-analysis. Reference [21].

Our literature search identified 1816 publications. Finally, a total of 12 studies were included in the analysis after assessing eligibility. These include four RCTs, one post hoc subgroup analysis, one follow-up study of an RCT, and six cohort studies (two prospective and four retrospective studies) [8-14,16-20]. Figure 1 shows the PRISMA flow diagram.

The characteristics of the included studies are described in Table 1. The TRAPS two-year follow-up study by Pengo et al. [17] was included along with the TRAPS trial [9]. Patients who had an outcome event (n=8) during the two-year follow-up (between January 28, 2018, and January 28, 2020) did not have those events during the TRAPS trial (before January 28, 2018) [9,17].

**Table 1:** Characteristics of the included studies. EUDRA: European union drug regulatory authorities, TRAPS: trial of rivaroxaban in antiphospholipid syndrome; RCT: randomized controlled trial; APS: antiphospholipid syndrome; DOACs: direct oral anticoagulants; TE: thromboembolic events; VKAs: vitamin K antagonists; SVT: superficial vein thrombosis; DVT: deep vein thrombosis; TIA: transient ischemic attack; MI: myocardial infarction; VTE: venous thromboembolism; PE: pulmonary embolism; ETP: endogenous thrombin potential; RAPS: rivaroxaban versus warfarin to treat patients with thrombotic antiphospholipid syndrome, with or without systemic lupus erythematosus; ASTRO-APS: apixaban for secondary prevention of thromboembolism among patients with antiphospholipid syndrome. References [8-14,16-20].

S. no.	Study name/author name (reference]	Pub year	Study design	Study population	Patients (n)	Intervention	Outcomes	Follow-up (months)
1	EUDRA-2010-019764-36/Ordi-Ros et al. [8]	2019	Phase III RCT, multicenter	Adult APS patients with arterial or venous thrombosis receiving acenocoumarol	190	Rivaroxaban versus VKA	Primary: New thrombotic events and major bleeding. Secondary: Time to thrombosis, type of thrombosis, changes in biomarker levels, cardiovascular death, and non-major bleeding	36
									2	TRAPS/Pengo et al. [9]	2018	Phase III RCT, multicenter	Adult triple-positive (high-risk) APS patients with a history of thrombosis	120	Rivaroxaban versus warfarin	Primary: TE events, major bleeding, and vascular death. Secondary: Any single type of TE event and all-cause mortality	18
3	RE-COVER I/RECOVER- II/RE-MEDY/Goldhaber et al. [10]	2016	Post hoc subgroup analysis of the three Phase III RCTs (RE-COVER I, RE-COVER II, RE-MEDY)	Adults with objectively confirmed, symptomatic proximal DVT or PE (RE-COVER, RE-COVER II). Adults with objectively confirmed, symptomatic DVT or PE treated with approved anticoagulant for 3-12 months or with dabigatran in RE-COVER I or RE-COVER II (RE-MEDY)	151	Dabigatran versus warfarin	Primary: Recurrent objectively confirmed, symptomatic VTE or death associated with VTE. Secondary: Major bleeding, clinically relevant non-major bleeding, all bleeding events	6 (RE-COVER I/RE-COVER II). 6-36 (RE-MEDY)									
									4	RAPS/Cohen et al. [11]	2016	Phase II/III RCT, multicenter	Adult thrombotic APS patients with previous venous TE on standard intensity warfarin for at least three months	116	Rivaroxaban versus warfarin	Primary: Percentage change in ETP from randomization to day 42. Secondary: Occurrence of TE up to 210 days after randomization, thrombin generation, serious adverse events, and bleeding events	7
5	Sato et al. [12]	2019	Retrospective cohort	Adult APS with prior history of thrombosis	54	Rivaroxaban/edoxaban/apixaban versus warfarin	Primary: Event-free survival for five years (recurrence of arterial/venous thrombosis and severe bleeding requiring hospitalization and/or transfusion).	60									
									6	Martinelli et al. [13]	2018	Prospective cohort, single center	Adult APS patients with a history of venous thrombosis	28	Rivaroxaban versus VKA	Primary: Recurrence of thrombosis. Secondary: Major bleeding and clinically relevant non-major bleeding.	21.9
7	Malec et al. [14]	2019	Prospective cohort, single center	Adult patients with APS	176	Rivaroxaban/apixaban/dabigatran versus warfarin	Primary: Symptomatic TE events (venous or arterial), PE, SVT, stroke, TIA, MI. Secondary: Major bleeding, clinically relevant non-major bleeding.	51									
									8	ASTRO-APS/Woller et al. [16]	2021	Phase IV RCT, multicenter	Adult thrombotic APS patients receiving some form of anticoagulation	48	Apixaban versus warfarin	Primary: Rate of thrombosis and vascular death. Rate of major and clinically relevant non-major bleeding. Secondary: Net clinical benefit	12
9	TRAPS (two-year follow-up)/Pengo et al. [17]	2020	Phase III RCT, multicenter	Adult triple-positive APS patients with a history of thrombosis	115	Rivaroxaban/dabigatran versus warfarin	Recurrent thromboembolic events	24									
									10	Williams et al. [18]	2021	Retrospective cohort, single center	Isolated or double-positive APS patients treated with DOACs (apixaban, dabigatran, rivaroxaban) or warfarin	96	Apixaban/dabigatran/rivaroxaban versus warfarin	Primary: Recurrent TE events (arterial and venous) Secondary: Major bleeding events	72
11	Franke et al. [19]	2021	Retrospective cohort, single center	Adult APS patients with prior history of thrombosis receiving warfarin or DOACs	200	Rivaroxaban/apixaban/dabigatran/edoxaban versus warfarin	Recurrent thromboembolic events	16-32 (Median)									
									12	Liu et al. [20]	2022	Retrospective cohort, single center	Adults with single-positive APS on anticoagulation with warfarin or an anti-Xa DOAC for venous or arterial thromboembolism	143	Rivaroxaban/apixaban versus warfarin	Primary: First recurrent thromboembolic event (VTE or arterial thrombosis). Secondary: Major bleeding events	12

A total of 1437 adults with APS followed over a mean weighted period of 28.5 months (ranging between 6 and 72 months) were included in this meta-analysis. Of these, 634 constituted the DOACs arm (44%), and 803 constituted the VKAs group (56%). The mean age was similar in both groups (48.4 years for DOACs and 48.5 years for VKAs; p=0.95). Females constituted 61.3% of the DOACs group and 56.2% of the VKAs arm. The mean body mass index (BMI) was also comparable between the groups (28.6 versus 28.4, respectively; p=0.88).

**Figure 2:**
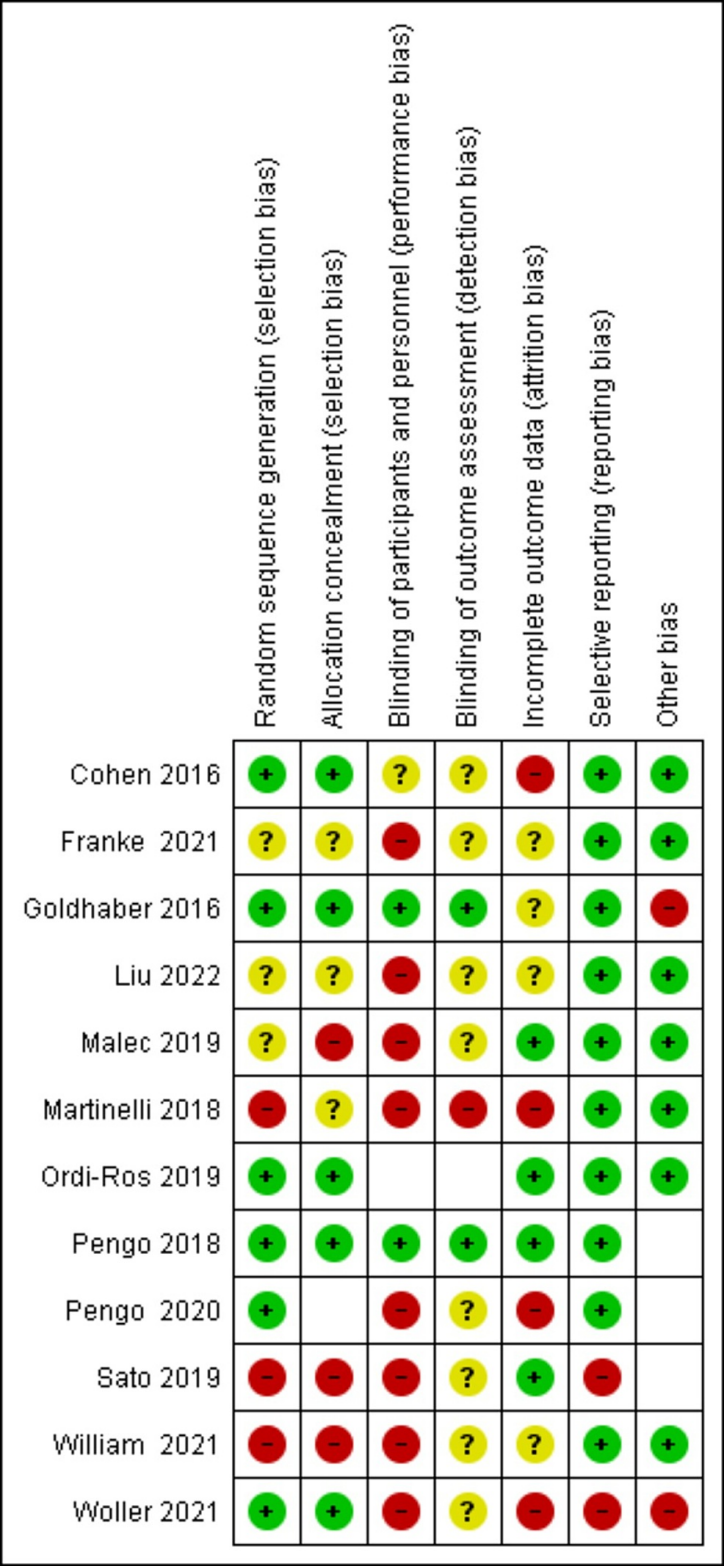
Risk of bias summary: review authors' judgments about each risk of bias item for each included study. References [8-14,16-20].

A summary of the risk of bias for each individual study is depicted in Figure 2. 

Outcomes

**Figure 3:**
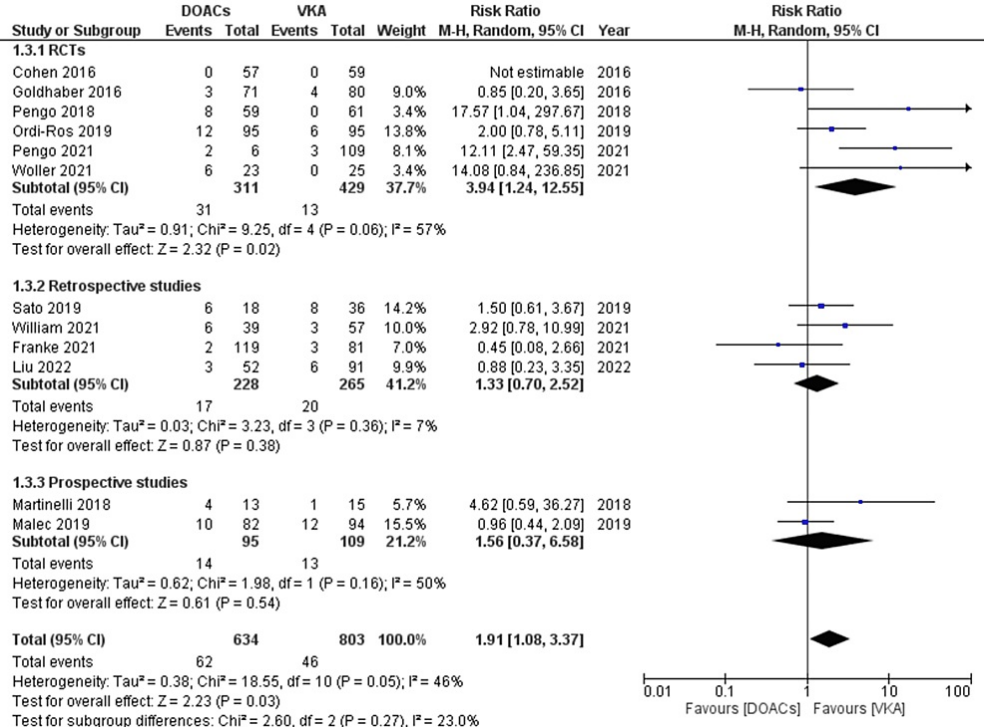
Forest plot of recurrent thromboembolic events with DOACs versus VKAs based on study design. RCT: randomized controlled trial; DOACs: direct oral anticoagulants; VKA: vitamin K antagonists; CI: confidence interval. References [8-14,16-20].

The risk of all recurrent TE events in APS patients while on DOACs compared to VKAs: A total of 108 patients (7.51%) developed recurrent TE events. Of these, 55 were arterial events (50.9%), and 53 were venous (49.0%). In the DOACs group, 62 out of 634 patients (9.7%) developed recurrent thrombosis; 38 (61.2%) were arterial and 24 (38.7%) venous. In the VKAs group, out of 803 patients, 46 (5.72%) suffered recurrent thromboembolism with a predominance of venous events (63%). The primary outcome of recurrent TE events was significantly higher in the DOACs arm compared to the VKAs (RR 1.91, 95% CI 1.08-3.37; p=0.03; I^2^=46%; 12 studies). Though no statistically significant differences were observed between RCTs, and retrospective and prospective studies, the magnitude of the risk was superior in RCTs (RR 3.94 95% CI 1.24-12.55; p=0.02; I^2^=57%) (Figure 3).

**Figure 4:**
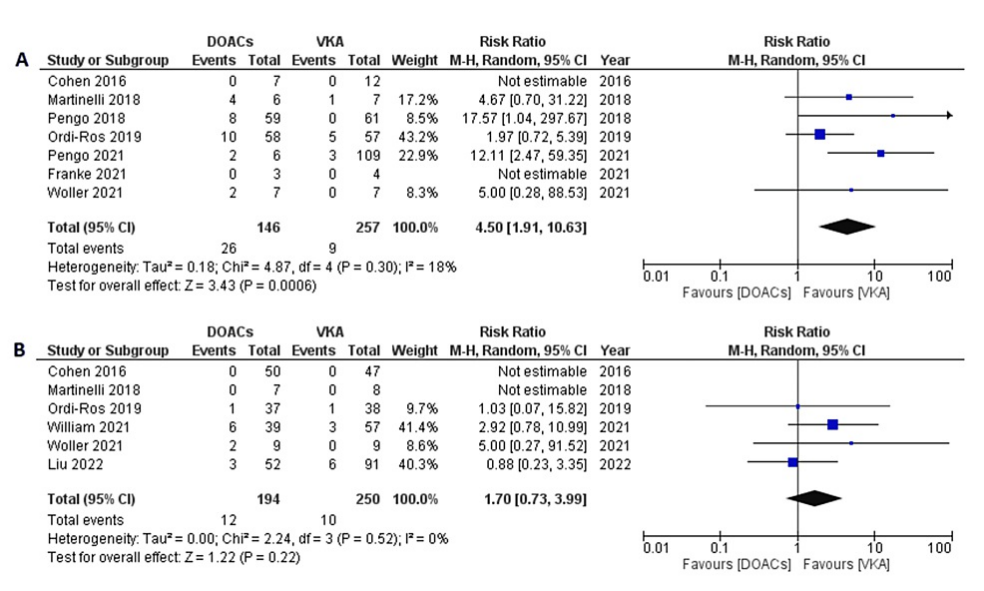
Forest plot of recurrent thromboembolic events with DOACs versus VKAs in (A) triple-positive (high-risk) APS and (B) isolated or double-positive APS. DOACs: direct oral anticoagulants; VKA: vitamin K antagonists; CI: confidence interval. References: [8-14,16-20].

When the subgroup of patients with triple-positive (high-risk) APS were analyzed separately, a higher magnitude of risk for recurrent thrombosis was observed in the DOACs arm (RR 4.50, 95% CI 1.91-10.63; p=0.0006; I^2^=18%; seven studies) (Figure 4A). The subgroup with double or isolated aPL also tended to develop increased risk; however, they did not reach statistical significance (RR 1.70, 95% CI 0.73-3.99; p=0.22; I^2^=0%; six studies) (Figure 4B). 

**Figure 5:**
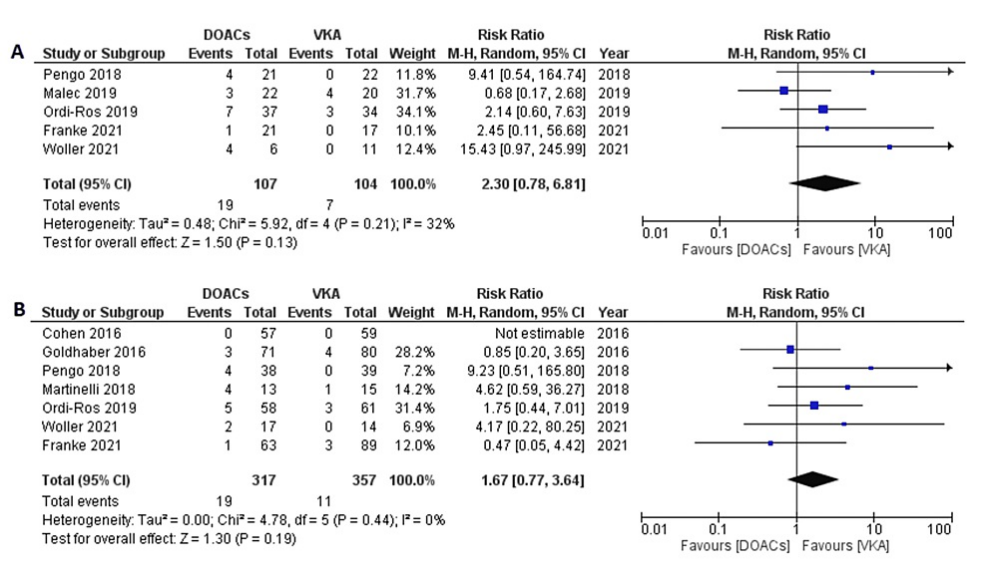
Forest plot of recurrent thromboembolic events with DOACs versus VKAs in patients with (A) previous arterial events and (B) previous venous events. DOACs: direct oral anticoagulants; VKA: vitamin K antagonists; CI: confidence interval. References: [8-14,16-20].

In patients with previous arterial thrombosis, the RR of recurrent TE with DOACs was 2.30 (95% CI 0.78-6.81; p=0.13; I^2^=32%) (Figure 5A). In patients with previous venous thrombosis only, RR was 1.67 (95% CI 0.77-3.64; p=0.19; I^2^=0%). (Figure 5B).

**Figure 6:**
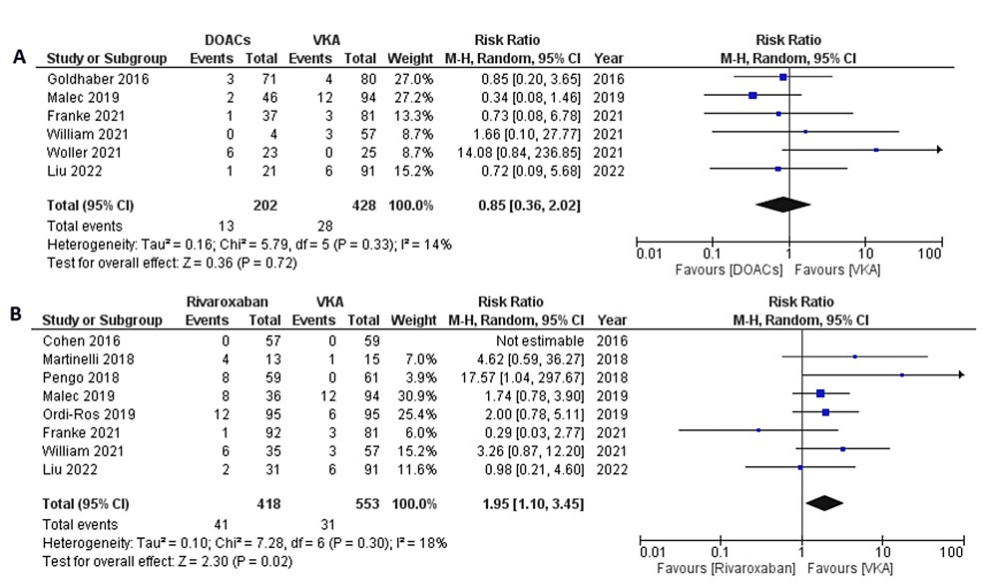
Forest plot of recurrent thromboembolic events with (A) DOACs other than rivaroxaban (apixaban, dabigatran, or edoxaban) versus VKAs and (B) rivaroxaban versus VKAs. DOACs: direct oral anticoagulants; VKA: vitamin K antagonists; CI: confidence interval. References: [8-14,16-20].

In the subgroup of patients treated with DOACs other than rivaroxaban, a slight decrease in risk was observed compared to VKAs but was not statistically significant (RR 0.85, 95% CI 0.36-2.02; p=0.72; I^2^=14%) (Figure 6A). However, the risk doubled in the subgroup that was treated exclusively with rivaroxaban compared to VKAs (RR 1.95, 95% CI 1.10-3.45; p=0.02; I^2^=18%) (Figure 6B).

**Figure 7:**
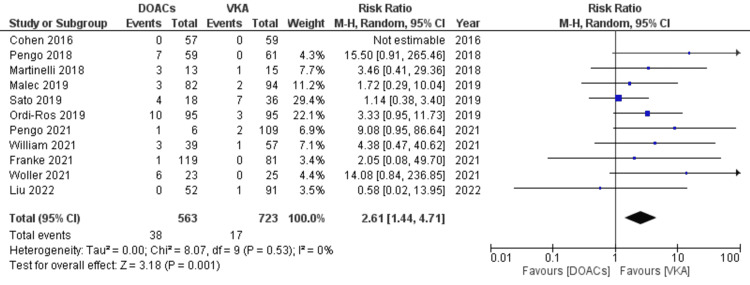
Forest plot of recurrent arterial events with DOACs versus VKAs. DOACs: direct oral anticoagulants; VKA: vitamin K antagonists; CI: confidence interval. References: [8-14,16-20].

Risk of arterial events in APS patients while on DOACs compared to VKAs: As previously indicated, the outcome of all TE events is a composite of arterial and venous outcomes. The DOACs arm had an increased risk of developing arterial events compared to VKAs (RR 2.61, 95% CI 1.44-4.71; p=0.001; I^2^=0%; 12 studies) (Figure 7). The risk of recurrent arterial thrombosis among the subgroup of patients receiving concomitant antiplatelet therapy was also analyzed. Two events were reported in the DOACs group (n=30) versus one in the VKAs arm (n=43). Although a trend for increased risk was observed in the DOACs arm, statistical significance was not achieved. 

**Figure 8:**
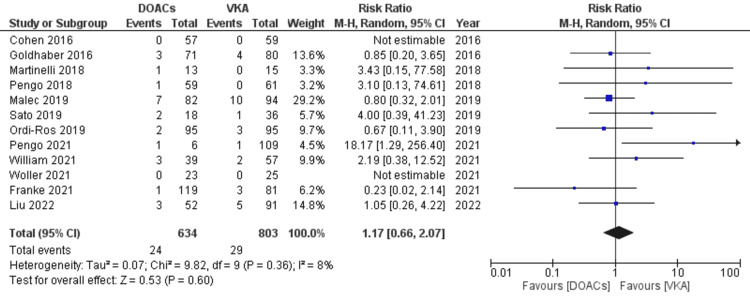
Forest plot of recurrent venous events with DOACs versus VKAs. DOACs: direct oral anticoagulants; VKA: vitamin K antagonists; CI: confidence interval. References: [8-14,16-20].

Risk of venous events in APS patients while on DOACs compared to VKAs: there was no statistically significant increase in risk was observed in the DOACs group compared to VKAs (RR 1.17, 95% CI 0.66-2.07; p=0.60; I^2^=8%; 12 studies) (Figure 8).

**Figure 9:**
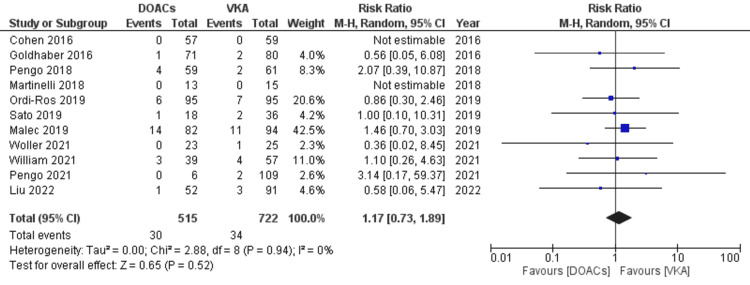
Forest plot of major bleeding events with DOACs versus VKAs. DOACs: direct oral anticoagulants; VKA: vitamin K antagonists; CI: confidence interval. References: [8-14,16-20].

The risk of major bleeding, all bleeding, and all-cause mortality in APS patients while on DOACs is compared to VKA: 30 of 515 patients (5.8%) had major bleeding events in the DOACs arm compared to 34 of 722 (4.7%) in the VKAs arm (RR 1.17, 95% CI 0.73-1.89; p=0.52; I^2^=0%; 11 studies) (Figure 9). 

**Figure 10:**
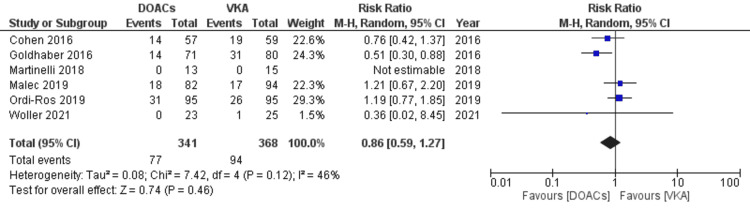
Forest plot of all bleeding events with DOACs versus VKAs. DOACs: direct oral anticoagulants; VKA: vitamin K antagonists; CI: confidence interval. References: [8-14,16-20].

Though a decreased risk for all bleeding events was observed in the DOACs arm compared to VKAs, the results were statistically non-significant (RR 0.86, 95% CI 0.59-1.27; p=0.46; I^2^=46%; six studies) (Figure 10).

**Figure 11:**
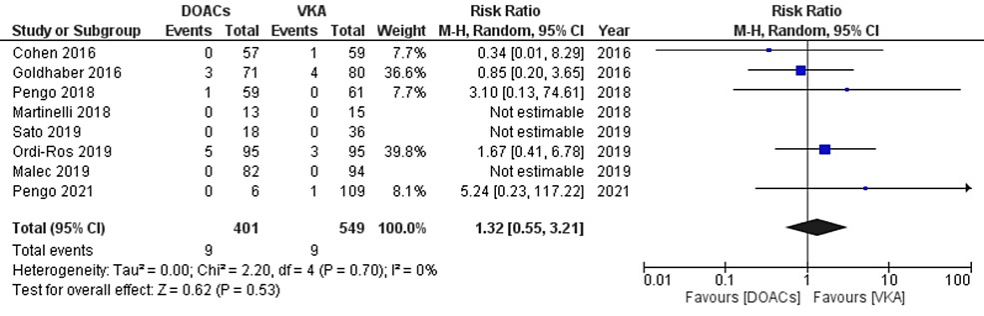
Forest plot of mortality events with DOACs versus VKAs. DOACs: direct oral anticoagulants; VKA: vitamin K antagonists; CI: confidence interval. References: [8-14,16-20].

Likewise, with regards to the risk of all-cause mortality, no statistically significant increase was observed in DOACs compared to VKAs. (RR 1.32, 95% CI 0.55-3.21; p=0.53; I^2^=0%; eight studies) (Figure 11).

Discussion

Our meta-analysis clearly shows that the use of DOACs in APS patients is associated with double the risk of recurrent thromboembolism when compared to VKAs. More specifically, we observed a significant increase in risk for arterial events (stroke/TIA and MI) but not venous thromboembolism (DVT, PE). Several studies reported recurrent arterial events in patients with previous arterial thrombosis [16,19]. We hence sought to analyze the risk of recurrent thrombosis according to the index thrombotic event. In our subgroup analysis of patients with prior arterial thrombosis, the risk of recurrent thrombosis was higher in the DOACs arm compared to VKAs, but the results were not statistically significant. A similar pattern was observed in the subgroup with prior venous thromboembolism. Triple-positivity is indicative of a major risk factor for thrombosis and obstetric complications [22]. In the TRAPS RCT, where only patients with triple-positivity were included, recurrent arterial thrombotic events were higher in the rivaroxaban arm (7 of 59) compared to the warfarin (0 of 61) [9]. In addition, in a retrospective cohort study by Williams et al. which excluded triple-positive patients, the proportion of patients with recurrent TE was three times higher in the DOACs group (6 of 39) compared to the warfarin group (3 of 57); however, this was not statistically significant (p=0.15) [18]. In our subgroup analysis of triple-positive (high-risk) APS patients, the risk of recurrent thrombosis was four times higher with DOACs compared to VKAs. The increased risk did not reach statistical significance in patients with isolated or double-positivity. In animal models of APS, platelets were shown to play a major role in thrombus formation within the arterial circulation [23]. The use of low-dose aspirin is hence considered in APS patients with a history of arterial thrombosis [6]. In our subgroup analysis, we attempted to determine if the risk of recurrent arterial thrombosis in the DOACs group changed with the concomitant use of antiplatelet therapy. However, no conclusions could be drawn due to the small sample size of patients who took aspirin or other antiplatelet therapies in these studies.

The risk of major bleeding and mortality was increased in patients treated with DOACs. However, statistical significance was not achieved for these outcomes. The risk of all bleeding events showed a trend favoring DOACs but failed to reach statistical significance. Finally, in a majority of studies, rivaroxaban was the most commonly used agent in the DOACs arm; very few studies had patients treated with apixaban, dabigatran, or edoxaban. In our subgroup analysis, DOACs other than rivaroxaban showed a slight decrease in the risk of recurrent thrombosis compared to VKAs, but statistical significance was not achieved, likely due to the small sample size. However, in patients on rivaroxaban, the thromboembolic risk doubled compared to VKAs. This data may suggest that while rivaroxaban increases the TE risk, other drugs in the DOACs group might be substituted for VKAs in the prevention of recurrent TE events in APS patients. Nonetheless, no definitive conclusions can be made regarding the safety and efficacy of DOACs other than rivaroxaban (i.e., apixaban, dabigatran, or edoxaban) due to the small number of patients treated with these drugs in the DOACs arm of the included studies. Further data from RCTs and/or observational studies will help clarify this risk or benefit.

Although not completely understood, several hypotheses have been made regarding the rationale behind higher thrombotic risk with DOACs. Unlike warfarin, which reduces functional levels of all vitamin K-dependent clotting factors, DOACs control thrombogenesis by selectively inhibiting factors Xa or IIa [11]. All phases of thrombin generation are equally affected by warfarin, while DOACs mainly affect the initiation and propagation phases. This leads to a delay in the formation of the prothrombinase complex, lengthening the lag time and the time to peak thrombin generation with DOACs [11]. Additionally, aPL antibodies were also found to increase the lag time and time to peak thrombin generation, leading to platelet activation and fibrinolysis impairment [11,15,23]. Moreover, DOACs exert their effect in a dose-dependent manner. Suboptimal dosing, insufficient drug concentrations, or a short half-life might also contribute to the lack of efficacy with DOACs [24]. Most of the available studies had patients on 15-20 mg rivaroxaban/day. These doses of DOACs may not provide sufficient protection against thrombosis in patients who require high-intensity anticoagulation [25]. However, further testing in adequately powered clinical trials would be necessary to determine the risks or benefits of dose intensification. Rivaroxaban for stroke patients with antiphospholipid syndrome (RISAPS) is an ongoing phase 2/3 RCT that aims to assess the efficacy of high-intensity rivaroxaban (15 mg twice daily) versus high-intensity warfarin (INR 3.5) in APS patients with a history of stroke or other ischemic brain manifestations (NCT03684564).

There are, however, certain limitations to the studies included in our analysis. Not all studies were clear about whether patients were recruited based on the Sapporo criteria [1]. Positive lupus anticoagulant is associated with a higher risk of thrombosis [26]. Most of the studies lacked identification of associated antiphospholipid antibodies in patients with higher thrombosis risk. Studies also had variable follow-up times ranging from 6 to 72 months. It can be presumed that in studies with shorter follow-up, the number of thromboembolic events would be much higher if the patients were followed for a longer duration resulting in a higher risk for recurrent TE events than reported. Moreover, several confounding factors such as cardiovascular risk factors (smoking, hypertension, diabetes mellitus, dyslipidemia), coexisting hereditary thrombophilia, concomitant autoimmune conditions such as systemic lupus erythematosus, and other hypercoagulable states might play a role in increasing the thrombosis risk. Very few studies looked for these characteristics in patients with recurrent thromboembolic events. Thus, further studies without these limitations would be noteworthy.

## Conclusions

To conclude, our updated meta-analysis reaffirms that the use of DOACs, particularly rivaroxaban poses an increased risk of arterial thrombosis in high-risk (triple-positive) APS patients and is thus not effective in preventing recurrent TE events in these patients. Further studies are warranted to confirm the safety and efficacy of DOACs other than rivaroxaban in the prevention of venous thromboembolism in low-risk APS patients. As such, until further evidence is available, warfarin should remain the drug of choice. 
